# Multilayered stable 2D nano-sheets of Ti_2_NT_x_ MXene: synthesis, characterization, and anticancer activity

**DOI:** 10.1186/s12951-019-0545-4

**Published:** 2019-11-11

**Authors:** A. Szuplewska, A. Rozmysłowska-Wojciechowska, S. Poźniak, T. Wojciechowski, M. Birowska, M. Popielski, M. Chudy, W. Ziemkowska, L. Chlubny, D. Moszczyńska, A. Olszyna, J. A. Majewski, A. M. Jastrzębska

**Affiliations:** 1 Faculty of Chemistry, Warsaw University of Technology, Noakowskiego 3, 00-664 Warsaw, Poland; 2 Faculty of Materials Science and Engineering, Warsaw University of Technology, Wołoska 141, 02-507 Warsaw, Poland; 3 Faculty of Physics, University of Warsaw, Pasteura 5, 00-092 Warsaw, Poland; 4 Faculty of Materials Science and Ceramics, AGH University of Science and Technology, Mickiewicza 30, 30-059 Kraków, Poland

**Keywords:** MXenes, Ti_2_N, Cytotoxicity in vitro, Stability, Mammalian cells, Anticancer properties

## Abstract

**Background:**

The biological activity of MXenes has been studied for several years because of their potential biomedical applications; however, investigations have so far been limited to 2D titanium carbides. Although monolayered Ti_2_NT_x_ MXene has been expected to have biological activity, experimental studies revealed significant difficulties due to obstacles to its synthesis, its low stability and its susceptibility to oxidation and decomposition.

**Results:**

In this paper, we report our theoretical calculations showing the higher likelihood of forming multilayered Ti_2_NT_x_ structures during the preparation process in comparison to single-layered structures. As a result of our experimental work, we successfully synthesized multilayered Ti_2_NT_x_ MXene that was suitable for biological studies by the etching of the Ti_2_AlN MAX phase and further delamination. The biocompatibility of Ti_2_NT_x_ MXene was evaluated in vitro towards human skin malignant melanoma cells, human immortalized keratinocytes, human breast cancer cells, and normal human mammary epithelial cells. Additionally, the potential mode of action of 2D Ti_2_NT_x_ was investigated using reactive oxygen tests as well as SEM observations. Our results indicated that multilayered 2D sheets of Ti_2_NT_x_ showed higher toxicity towards cancerous cell lines in comparison to normal ones. The decrease in cell viabilities was dose-dependent. The generation of reactive oxygen species as well as the internalization of the 2D sheets play a decisive role in the mechanisms of toxicity.

**Conclusions:**

We have shown that 2D Ti_2_NT_x_ in the form of multilayered nanoflakes exhibits fair stability and can be used for in vitro studies. These results show promise for its future applications in biotechnology and nanomedicine.

## Highlights

1. 2D multilayered nano-sheets of Ti_2_NT_x_ MXene were successfully obtained using classic etching and delamination
2. 2D Ti_2_NT_x_ showed higher toxicity towards cancerous cell lines (MCF-7, A365)
3. The decrease in cells viability was dose-dependent
4. 2D Ti_2_NT_x_ was not toxic towards non-malignant cells (MCF-10A, HaCaT)
5. ROS generation and internalization were identified mechanisms of toxicity

## Background

The discovery of graphene shifted the research on nanomaterials towards their two-dimensional (2D) homologs [1]. Since then, the dynamic development of this field of nanotechnology has progressed, and new members have joined the family of 2D materials. Within this family, several smaller groups can be distinguished, such as transition metal dichalcogenides [2], nitrides [3], di-transition metal sulfides, selenides and phosphides [4, 5], 2D oxides and hydroxides [6, 7], 2D metal–organic covalent frameworks [8], as well as Xenes [9]. The most recent members of this group are early transition metal carbides, nitrides, and carbonitrides called MXene phases. They were first discovered in 2011 during studies on the application of MAX phases in supercapacitors by Naguib, Barsoum, and Gogotsi from Drexel University, USA [10]. This new class of nanomaterials has so far demonstrated significant potential in many applications, such as energy storage [11], ceramic matrix composites [12–14], and macromolecules’ adsorption [15]. Other studies also revealed possibilities of their usage as biocides [16–18], in nanomedicine [19], or environmental remediation [20]. Due to the fact that the MXenes are developing much more dynamically in comparison to other 2D materials apart from graphene, they were considered as the most interesting and bringing the greatest innovation potential among their analogs [21].

At the same time, there is concern about the stability of some members of the MXene family, which can significantly affect their future use. The reason is that they are variable non-stoichiometric phases of their thermodynamically stable macroscopic counterparts (MAX phases). Their formulae are also written with a ‘T_x_’ termination, the concept of which is to indicate the presence of various functional groups present on their surface (i.e. –OH, –F, or =O) [22, 23]. In the great majority, the presence of OH groups indicates not only the hydrophilic properties of the MXenes’ surface, but also the process of surface oxidation [24]. Nevertheless, from the large number of MXenes phases, Ti_3_C_2_T_x_ and Ti_2_CT_x_ have so far proven to have relative stability in a number of applications [25]. There are also high hopes for the phases from the Nb-C or Mo-C [26] systems in relation to many applications. These phases are relatively easy to manufacture and to delaminate into 2D flakes.

On the other hand, there are MXene phases that are considered relatively unstable, such as Ti_2_NT_x_ [27] or Ti_4_N_3_T_x_ MXene [28]. The single-layered Ti_2_NT_x_ with ferromagnetic and partially metallic properties has been outlined as a result of theoretical research [27], whereas Ti_4_N_3_T_x_ has been recently successfully synthesized using molten fluoride salts [28]. Theoretical calculations, however, indicated that aqueous hydrofluoric acid (HF) solution cannot be successfully used for the aluminum etching and delamination of the precursor Ti_2_AlN MAX phase [29], due to the fact that single layered nitride MXenes possess poor stability in this solution. They should also possess very low values for the calculated cohesive energies of the hexagonal crystal structure, together with a stronger bonding of Al atoms [29, 30]. These values indicate a low stability for their single-layered structure, which is present only in some theoretical calculations. On the other hand, the Ti_2_N phase is expected to have intriguing properties as a half-metal and spin-gapless semiconductor in spintronic applications [31]. Researchers who were interested in the verification of these theories in an experimental manner have attempted to conduct syntheses of these materials. The first success in this endeavor belonged to Soundiraraju et al. [32], who carried out the process of KF/HCl etching of the Ti_2_AlN phase into Ti_2_NT_x_. Also, nitridation of the Ti_3_C_2_T_x_ MXene by Yang et al. [33] allowed N-doped 2D Ti_2_NT_x_ to be obtained. It should be noted that the desirable phase was present in up to 20.7 at.% in the final product. However, the Ti_2_NT_x_ obtained by nitridation was not assessed for potential cytotoxicity. In the next stage of the development of Ti_2_NT_x_ technology, the possibility of the preparation of 2D monolayers was explored by Tsai et al. [29]. Non-layered Ti_2_N with a work function of ∼ 4.75 eV was synthesized by N_2_ plasma immersion and was successfully applied as the anode material of lithium-ion batteries.

To sum up, while 2D sheets of Ti_3_C_2_T_x_ or Ti_2_CT_x_ MXenes have been already widely studied with respect to their cytotoxicity, investigations on 2D Ti_2_NT_x_ have not been focused on biologically-related studies. As a result, up to now 2D Ti_2_NT_x_ has been an ‘enigma’ in terms of its potential cytotoxicity.

**Fig. 1:**
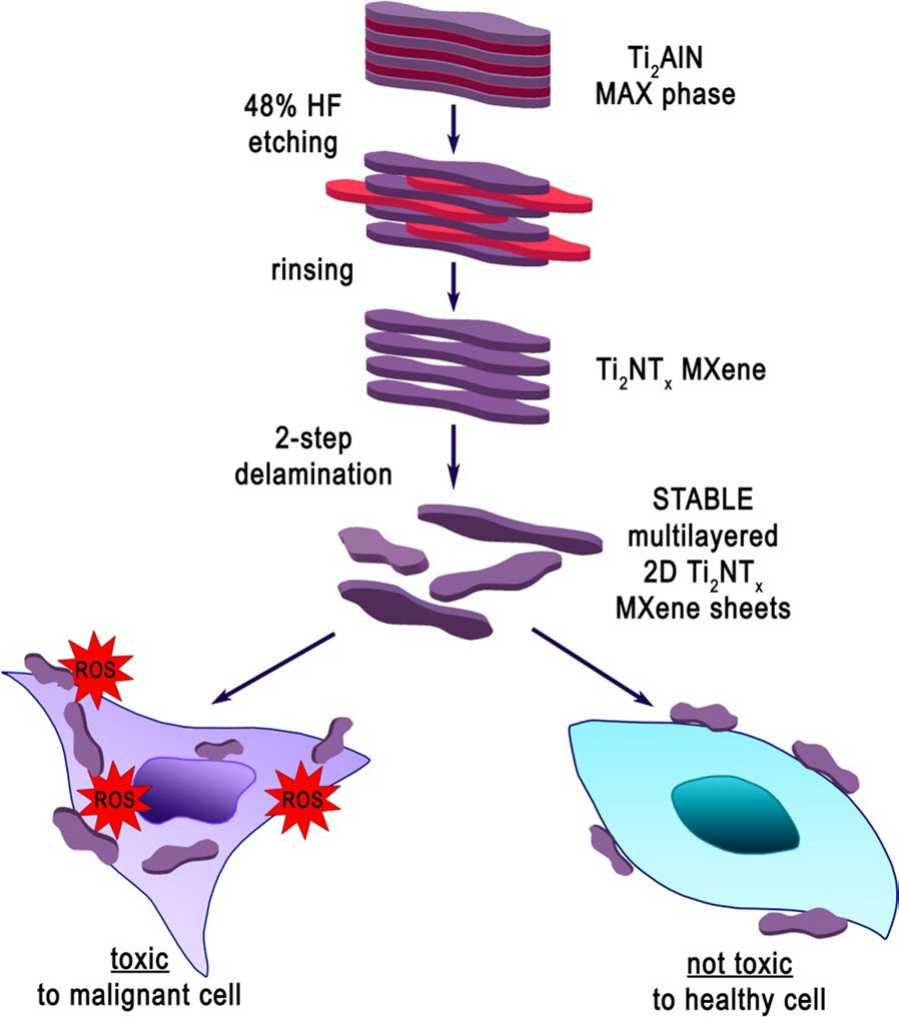
Schematic representation of the concept of the present study. The flowchart shows the synthesis process of the 2D Ti_2_NT_x_ MXene for obtaining stable multilayered 2D structures as well as the resulting interactions with human cells

In this study, we have successfully bypassed the problem of instability of the 2D Ti_2_NT_x_ MXene through obtaining multilayered nano-structures. Subsequently, we have undertaken the challenge of investigating the cytotoxic properties of the Ti_2_NT_x_ MXene phase after a delamination process into stable multilayered 2D flakes (Fig. 1). Herein, we present the first study on the synthesis of 2D sheets of Ti_2_NT_x_ MXene using classical aluminum etching of the Ti_2_AlN MAX phase and further delamination into multilayered flakes, as well as its resulting cytotoxicity and toxicity mechanisms. The results of the present study provide the principal knowledge currently unavailable regarding the toxicity and potential anticancer properties of delaminated 2D multilayered sheets of Ti_2_NT_x_ MXene.

## Materials and methods

### Synthesis

The starting Ti_2_AlN MAX phase was synthesized using the self-propagating high-temperature synthesis (SHS) technique in 1.5 atm of argon underpressure. As a substrates, 325 mesh (44 micron) titanium metal powder Ti-109 of 99.7% purity from Atlantic Equipment Engineers and the intermetallic material (TiAl) which was synthesized from elemental powders by SHS method initiated with thermal explosion, were used. The reaction equation was as follows:

1 $$2 {\text{TiAl}} + {\text{Ti}} + {\text{N}}_{ 2} = {\text{Ti}}_{ 2} {\text{AlN}} .$$

The MAX product was then initially crushed and then milled with the use of rotary-vibratory mill in isopropanol environment for 12 h. The ‘widia’ (WC–Co) balls were used as milling media.

The obtained powdered MAX phase was immersed for etching in concentrated 48% (v/v) hydrofluoric acid (HF, Sigma-Aldrich) for 24 h with continuous stirring at room temperature using a ratio of acid: powder of 10 cm^3^: 1 g. In the next step, the obtained material was subjected to sedimentation, washed 5× with distilled water and 5x with ethanol. The washed material was dried overnight at room temperature.

Delaminated 2D sheets of the Ti_2_NT_x_ were obtained using the protocol previously developed by us for obtaining multilayered 2D Ti_3_C_2_T_x_ and Ti_2_CT_x_ MXenes [15]. Briefly, it was a two-step process in which non-polar (hexane) and polar (isopropyl alcohol) solvents were used one after another with a ratio of 50 cm^3^ per 1 g of MXene powder. In the first step, the non-polar solvent hexane was used together with high-energy probe sonication (Vibra-Cell VCX750, 750Watt, 20 kHz, Sonics & Materials Inc.) on the prepared suspension for a total of 0.5 h. Subsequently, the powder was easily decanted, removed, and dried in the air. The next step involved tip sonication in isopropyl alcohol for a total of 15 min. The obtained suspension was centrifuged at 2500 rpm for 2 min. The obtained supernatant was removed, and the solid was dried for 6 h at room temperature and stored in argon at 5 °C for further use.

### Characterization

A scanning electron microscope (SEM-LEO 1530, Zeiss, USA) was employed for the morphology analysis of the Ti_2_AlN MAX phase, Ti_2_NT_x_ MXene after etching, as well as the multilayered 2D sheets of Ti_2_NT_x_ at an accelerating voltage of 5.0 kV. The sample was directly placed on sticky carbon tape and coated with a thin carbon layer. The multilayered 2D structure of Ti_2_NT_x_ MXene (placed on Cu-C mesh) was also examined in cross-section using a high-resolution transmission electron microscope (HREM, Philips CM 20). The atomic-scale of the Ti_2_N layers was revealed using Fourier transformation (FFT) together with subsequent reversed Fourier transformation (IFFT). The layered structure was also analyzed quantitatively by an intensity pattern taken perpendicularly to the plane formed by the individual Ti_2_N layers. This enabled analysis of the spacing between the brightest periods (i.e., d-spacing in nm). The selected area diffraction pattern (SADP) of the electrons was used for indication of the characteristic rings and identification of the measured interplanar distances for each Ti_2_N monolayer. The obtained results were analyzed for their similarity to the possible lattices of the space groups. The elemental composition of the multilayered 2D sheets of Ti_2_NT_x_ was checked using an energy-dispersive X-ray spectroscopy (EDX) unit coupled with a transmission electron microscope.

X-ray diffraction (XRD) was employed for the analysis of both the Ti_2_AlN MAX phase and the resulting Ti_2_NT_x_ MXene. The phase composition was examined in an X-ray diffractometer (Bruker D8 Advanced). The results were recorded within the 2ϴ angle range from 15 to 120° using CuKα radiation (λ  =  1.54 Å). The measuring step was Δ2θ  =  0.025° and the computation time was 3 s/step. The transmittance spectrum of the multilayered 2D sheets of Ti_2_NT_x_ was obtained using a UV–VIS spectrometer (Evolution 220 from Thermo Scientific) equipped with an integrating sphere (0.3 s of integration), in a range from 220 to 1100 nm, with a resolution of 1 nm, and a scanning speed of 200 nm min^−1^. The measurements were accompanied by a Tyndall test employing a sample suspended in double-distilled (DDW) water using red laser light.

Qualitative analysis of the presence of oxygen-containing functional groups on the surface of multilayered 2D sheets of Ti_2_NT_x_ was carried out using diffuse reflectance infrared Fourier transform spectroscopy (DRIFTS-FTIR, Nicolet iS5 Spectrometer from Thermo Scientific). The sample was mixed with dried KBr at a concentration of 2.5 wt %. Each spectrum was recorded with 60 scans, and OMNIC software from Thermo Fisher was used to analyze the obtained data.

The stability of the multilayered 2D sheets of Ti_2_NT_x_ was analyzed by dynamic light scattering (DLS) and zeta potential measurements (with the Smoluchowski model) using a NANO ZS ZEN 3500 analyzer (Malvern) equipped with a green laser light scattering detector, operating at an angle of 173º. All investigations were carried out in a double distilled water (DDW) suspension of multilayered 2D sheets of Ti_2_NT_x_ MXene phase at a concentration of 3.3∙10^−5^ g ml^−1^, at 25 °C. For the assessment of the stability of 2D sheets of Ti_2_NT_x_ in the most widely used in vitro cell culture media, the DDW was changed for the appropriate amounts of the culture media and incubated for t = 0 and t = 24 h. The analyzed media were Dulbecco’s Modified Eagle Media (DMEM), Roswell Park Memorial Institute (RPMI), and Minimum Essential Medium Eagle (MEME). The obtained results were presented as the mean value of zeta potential and hydrodynamic diameter, based on three independent experiments, respectively, with standard deviation.

### Theoretical calculations

Calculations were performed using spin polarized density functional theory (DFT) [34, 35] with the PBE exchange–correlation functional [36] as implemented in the VASP package [37, 38]. The electron–ion interaction was modeled by using projector augmented wave (PAW) pseudopotentials [39, 40], and a plane wave basis was set with a cutoff energy of 500 eV. The weak van der Waals forces were included by using the Grimme corrected approach (DFT + D2) [41]. The Brillouin zone integration of a k-point mesh of (24 × 24 × 2) in the Monkhorst–Pack sampling scheme [42] was used for the lateral (1 × 1) supercell and at least 17 Å of vacuum thickness. For the strongly localized 3d states of titanium, the DFT + U approach introduced by Dudarev et al. [43] was used. The effective on-site Coulomb and exchange parameters for the 3d states were set to U = 5 eV and J = 1 eV, respectively. All the structures were fully structurally optimized until the force on each atom and each component of the stress matrix was below 0.005 eV/Å and 2 kbar, respectively.

### Analysis of cytotoxicity in vitro

The biocompatibility of 2D Ti_2_NT_x_ MXene was evaluated towards human skin malignant melanoma cells (A375, ATCC), human immortalized keratinocytes (HaCaT, ThermoFisher), human breast cancer cells (MCF-7, ATCC), and normal human mammary epithelial cells (MCF-10A, ATCC). The cells’ viabilities were monitored after the incubation of cultures with increasing concentrations (0–500 mg L^−1^) of the studied material. The A375, HaCaT, and MCF-7 cell lines were cultured using complete Dulbecco’s Modified Eagle’s Medium (DMEM, Sigma-Aldrich) supplemented with 10% (v/v) fetal bovine serum (FBS), 1% (v/v) of penicillin and streptomycin, and 1% (v/v) of l-glutamine. The MCF-10A line was grown in F-12 DMEM containing 5% (v/v) horse serum, 10 μg mL^−1^ human insulin, 10 ng mL^−1^ epithelial growth factor, and 5 μg mL^−1^ hydrocortisone (Sigma-Aldrich). The cells were cultured under 5% CO_2_ at 37  °C and 95% humidity.

The tetrazolium dye 3-(4,5-dimethylthiazol-2-yl)-2,5-diphenyltetrazolium bromide (MTT) assay was applied as a tool for studying the influence of the tested material on cell cultures. All cell lines were seeded at a density of 1 × 10^4^ cells per well. Subsequently, the cells were incubated to assure their adhesion to the surface. The supernatant was then replaced with a series of dilutions containing various concentrations of MXene (100 μL per well), and the resulting mixtures were incubated for 24 h. Controls were run in the absence of MXene (the cell culture was incubated with the appropriate fresh medium only). Each experiment was conducted at least three times independently. After exposure to 2D Ti_2_NT_x_, the cells were washed three times with phosphate-buffered saline (PBS, Sigma-Aldrich) and treated with MTT (Sigma-Aldrich) solution (0.5 mg mL^−1^ in PBS; 100 μL per well). The cells were incubated with MTT for the next 4 h and protected from light. The supernatant was then carefully removed, and the formed violet formazan crystals were dissolved in dimethyl sulfoxide (DMSO, Sigma-Aldrich; 100 μL per well). The absorbance was measured at 570 nm, and the results were expressed as the percentage viability comparing to the controls.

### Analysis of the mechanism of action

In order to verify if the potential cytotoxicity of Ti_2_NT_x_ is the result of oxidative stress, the level of intracellular reactive oxygen species (ROS) was measured in the presence of the non-specific fluorescent dye, 2′,7′–dichlorofluorescein diacetate (DCF–DA, Sigma–Aldrich). After 24 h of exposure to MXene, the cells were washed three times with PBS and subsequently maintained in DCF–DA solution (20 μM in PBS; 100 μL per well) for 30 min in the dark. The supernatant was then replaced with 0.1% Triton-X solution (Sigma-Aldrich) (v/v) (100 μL per well). Cells were incubated with a solution of surfactant until lysis was observed (1 h). The fluorescence intensity was measured at 530 nm (excitation wavelength: 495 nm), and the results were expressed as a percentage of ROS compared to the controls.

Analysis of the cellular uptake of 2D Ti_2_NT_x_ sheets was performed using a scanning electron microscope (SEM) combined with an energy-selective backscattered (ESB) detector for the imaging of their presence inside or outside the cells. The cells derived from skin were seeded on the surface of Muscovite Mica rings (V1, Science Service) suitable for SEM observations and cultured under physiological conditions until cellular attachment was observed. Subsequently, the culture media were replaced with MXene suspensions in concentrations of either 62.5 μg mL^−1^ or 500 μg mL^−1^. Controls were run in the absence of MXene (the cell culture was incubated with fresh DMEM only). After 24 h of exposure to the tested nanomaterials, the cells were fixed in the presence of 3% (v/v) glutaraldehyde (Sigma-Aldrich) in PBS for 4 h at 0  °C. The cells were then washed three times with PBS (3  ×  10 min) and dehydrated using increasing concentrations of ethyl alcohol (Sigma-Aldrich), 50%–100% (v/v), for 5 min at each concentration. The samples were next coated with a thin carbon layer and were observed by SEM (LEO 1530, Zeiss, USA). The SEM observations were conducted at 2.0 kV, while ESB imaging was conducted at 12.0 kV.

## Results and discussion

### Results of characterization

**Fig. 2:**
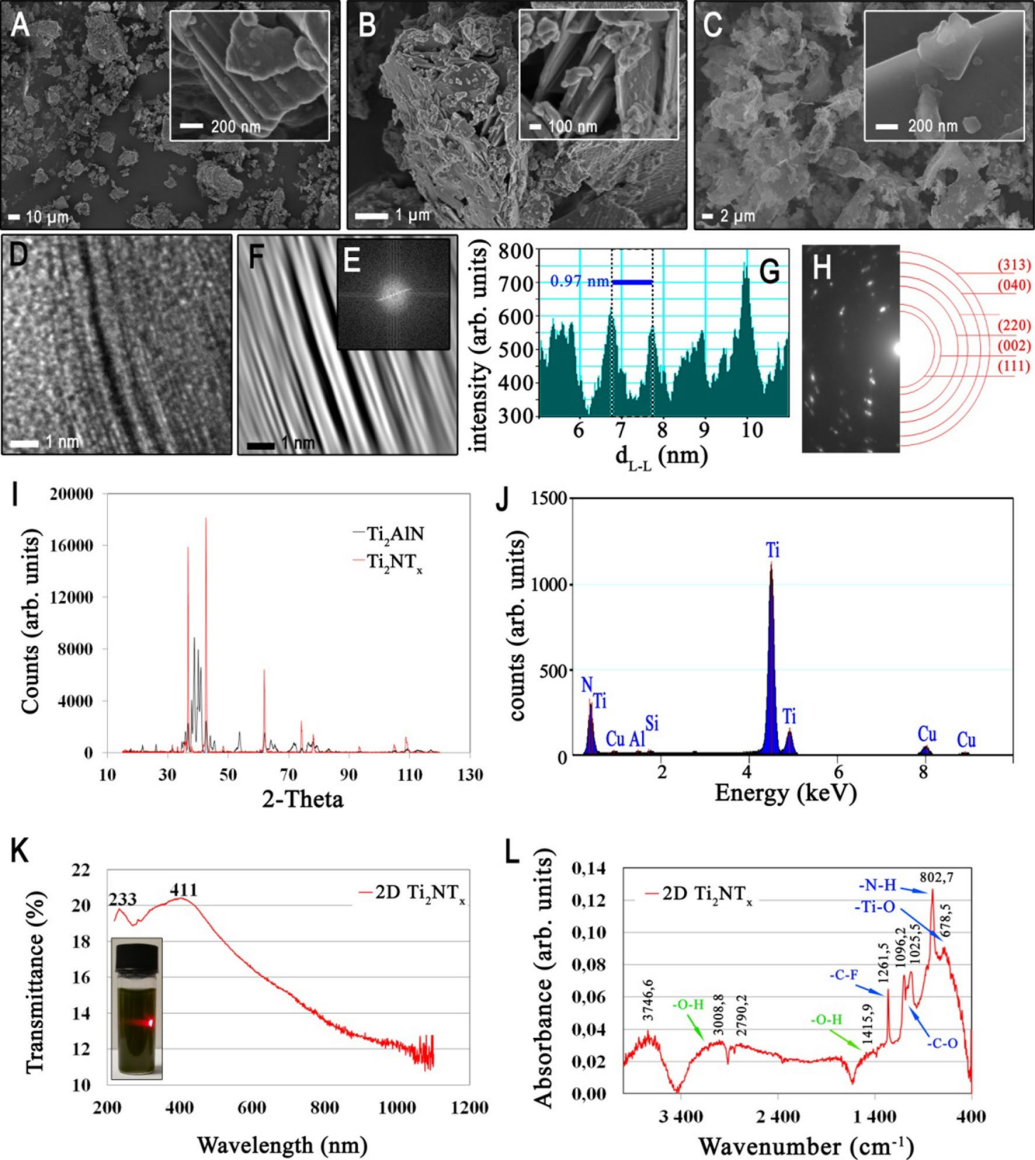
Characterization of the materials used in the study i.e.: Representative SEM images obtained for **a** Ti_2_AlN starting MAX phase, **b** Ti_2_N MXene after HF etching, and **c** 2D sheets of Ti_2_NT_x_ MXene after delamination; **d** Cross-sectional HREM image, **e** FFT image together with related **f** IFFT extraction of multilayered structure obtained for 2D sheets of Ti_2_NT_x_ MXene; **g** The intensity pattern revealing a period related to d-spacing of 0.97 nm; **h** SADP electron diffraction with indication of the characteristic rings for 2D Ti-N layers; **i** Comparison of the X-ray diffraction patterns obtained for: Ti_2_AlN MAX phase and resulting Ti_2_NT_x_ MXene; **j** EDX analysis, **k** UV–VIS spectrum accompanied by Tyndall test, as well as **l** FTIR spectrum of the Ti_2_NT_x_ MXene after delamination

Detailed characterization of the materials used in the study is presented in Fig. 2. The typical layered compact structure of the Ti_2_AlN MAX starting phase is shown in Fig. 2a. This MAX phase was used for HF etching to obtain Ti_2_N MXene (Fig. 2b), which represents the typical morphology characteristic for MXenes [15, 16]. However, it can be noted that the structure after HF etching is far from ideal. Each regular structure was broken into smaller pieces, which in turn can positively influence a more effective delamination process. After HF etching, the MXene was subjected to a two-step delamination process, which allowed 2D sheets of Ti_2_NT_x_ MXene (Fig. 2c) to be obtained. The individual 2D Ti_2_NT_x_ layers possessed non-regular shapes and clearly had 2D morphology. It can also be noted that the reduction in the sizes of the flakes before and after delamination was of one order of magnitude. We thus finally obtained the 2D nanomaterial. Subsequently, the layered structure of the delaminated 2D Ti_2_NT_x_ flakes was analyzed in detail. Figure 2D presents a cross-sectional HREM image which clearly shows their multilayered features. The FFT image together with the related IFFT extraction of the multilayered structure of the obtained 2D sheets of Ti_2_NT_x_ MXene are presented in Fig. 2e and f, respectively. The obtained results confirmed the typical periodical intensity in which bright layers corresponded to individual Ti_2_N monolayers, whereas dark layers related to the apparently empty space occupied by the T_x_ species. Analysis of the intensity pattern for Ti_2_NT_x_ revealed a period related to d-spacing of 0.97 nm (Fig. 2g). It was also important to describe the type of lattice in which the Ti_2_N monolayers were organized. Unexpectedly, the SADP electron diffraction revealed the presence of characteristic rings for 2D Ti-N layers (Fig. 2h) [29, 30]. The X-ray diffraction patterns obtained for the Ti_2_AlN MAX phase and the resulting Ti_2_NT_x_ MXene are presented for comparison with each other in Fig. 2i. The patterns are different, especially in the 2-Theta range of 30–50°, showing significant changes in the crystal structure of the MAX phase after aluminum removal. The subsequent EDX analysis of the 2D Ti_2_NT_x_ MXene (Fig. 2j) additionally confirmed the effective removal of the aluminum, as well as revealing the presence of expected elements such as titanium and nitrogen. Furthermore, the nanocolloidal water dispersion of the 2D Ti_2_NT_x_ MXene was first confirmed as a nano-colloidal feature by the Tyndall test (see insert in Fig. 2k) and then was analyzed using UV–VIS and FTIR spectroscopy. The obtained UV–VIS transmittance spectrum is presented in Fig. 2k. Two maxima were seen at 233 and 411 nm, which corresponded to the violet range of the VIS spectrum, as well as the appearance of the dark ‘khaki’ color of the 2D Ti_2_NT_x_ MXene dispersion. FTIR analysis was then performed to determine the relationship of the specific signals corresponding to the oxygen and nitrogen connections. The FTIR spectrum of the 2D Ti_2_NT_x_ MXene after delamination is presented in Fig. 2l. It should be noted that the intensity of the asymmetric vibrations of the –OH peaks at ca. 3100 and 1400 cm^−1^ was negligible, which indicated a deficiency in the Ti–OH bonding. Instead, a relatively high absorption peak was present at 679 cm^−1^, which corresponded to the majority of the Ti–O bonds present on the surface of the 2D Ti_2_NT_x_ MXene. The other types of vibration present in the analyzed sample were –N–H, located at ca. 679 cm^−1^, as well as -C-F (located at ca. 1262 cm^−1^). The presence of –N–H bonding related to the susceptibility of the nitrogen to form amine connections, whereas the presence of fluorine related to the use of hydrofluoric acid in the MXene synthesis process.

### Theoretical calculations results

It has been theoretically shown that functionalization of MXene monolayers stabilizes them with respect to their pristine Ti_n+1_N_n_T_x_ counterparts. In particular, oxygen functionalization exhibits the lowest cohesive energy compared to –F or –OH functionalized groups [44]. It is worth noting that stable structures after functionalization were also predicted theoretically for many other materials, e.g. carbon-like systems [45, 46].

**Fig. 3:**
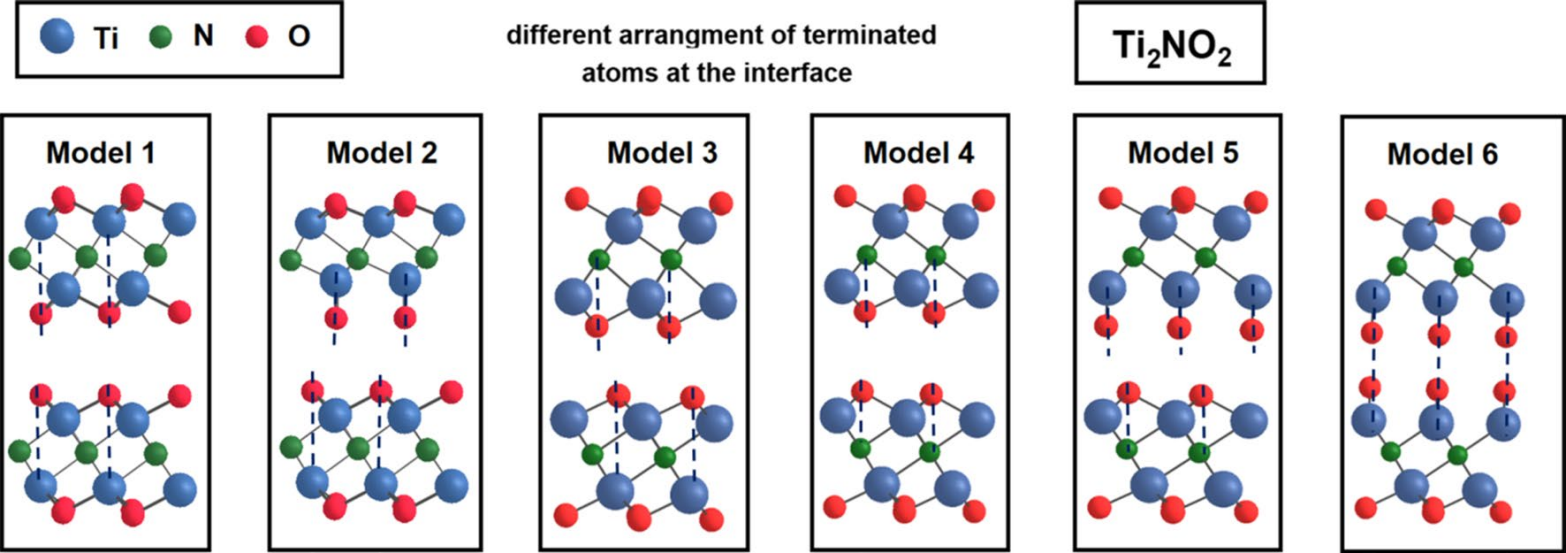
The possible arrangements of the oxygen atoms for two adjacent Ti_2_N layers. Model 1 is the most stable one from all the configurations considered here

First, on the basis of the experimental results, we assumed that the T_x_ termination in Ti_2_NT_x_ MXene was O_2_. Subsequently, we checked the different possible arrangements of the oxygen atoms at the interface between two layers as presented in Fig. 3. The most stable configuration was model 1 (see Fig. 3 and Table 1), in which each of the monolayers constituting the bilayer had the same arrangement of the oxygen atoms at the surface. Namely, the oxygen atoms were vertically aligned above the Ti atoms, which was consistent with the most stable termination of the monolayer of Ti_2_NO_2_ reported previously [43]. Furthermore, in our studies, only model 1 was taken into account. We fully optimized the position of atoms and the lateral size of the supercell consisting of one (1L) to five (5L) layers. Our studies revealed that the lateral lattice constant did not depend on the number of the layers, and thus it was fixed. Our optimized lattice parameter was equal to 3.082 Å, and it was slightly greater than the previously reported value of 3.00 Å for the Ti_2_NO_2_ monolayer with the PBE approach [47].

**Table 1:** Structural parameters of n-layers of Ti_2_NO_2_. Methods of computation: GGA + U(U = 5 eV, J = 1 eV), Grimme correction (DFT + D2)

Structure	Δ*E*	*a* [Å]
Model 1	0.000	3.082
Model 2	2.420	3.203
Model 3	0.514	3.054
Model 4	1.069	3.028
Model 5	–	–
Model 6	–	–

In the case of Model 5 and 6, the structures disintegrated into smaller structures, thus, no values in the table are provided

Subsequently, the same size supercell was used for each of the structures, with a vertical lattice constant equal to 51 Å, which was equal to the thickness of five layers of Ti_2_NO_2_ and 17 Å of vacuum. It should be noted that we also carefully checked the results for the different vertical sizes of the supercell, in which the thickness of the vacuum layer was fixed at 17 Å for each of the calculated structures. The difference in the interlayer binding energies between the former and latter cases was comparable within a few hundredths of meV/Å^2^. Our results indicated that the layer thicknesses did not depend on the number of layers constituting the Ti_2_NO_2_ MXene material (see Tab. 2.), whereas the interlayer distance slightly decreased as the number of the layers increased. The reduction of the interlayer distance reflected the enhancement of the attractive forces between the layers, and thus the decrease of the interlayer binding energy (stronger binding) was visible for an increasing number of layers.

**Table 2:** Structural parameters of n-layers of Ti_2_NO_2_. Methods of computation: GGA + U(U = 5 eV, J = 1 eV), Grimme correction (DFT + D2)

*n* layers	*d*_interlayer_	*d*_layer_ [meV/atom]
Structural parameters of n-layered Ti_2_NO_2_		
1L	–	2.595
2L	5.022	2.594
3L	4.978	2.594
4L	4.957	2.593
5L	4.960	2.593
BULK	4.944	2.591

**Fig. 4:**
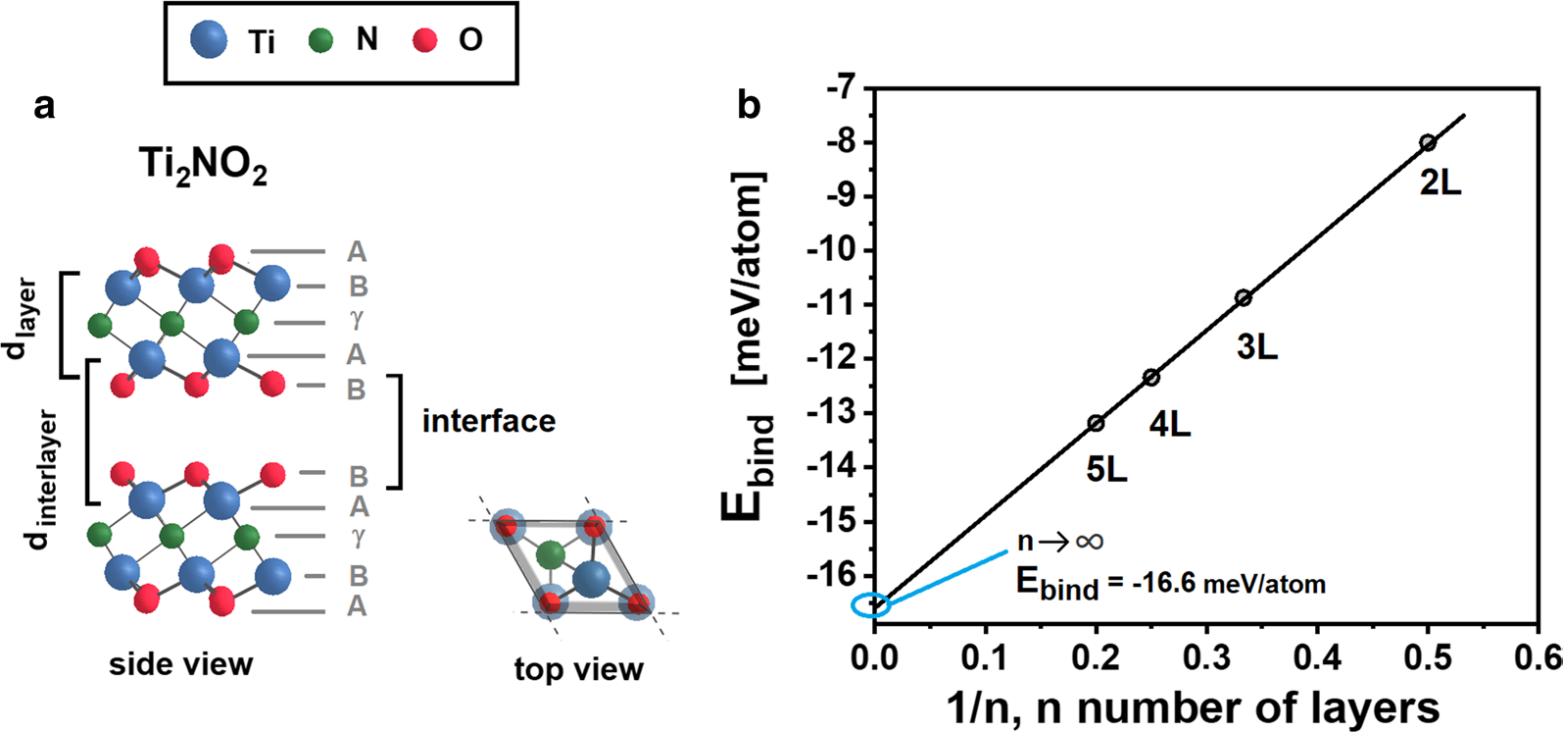
**a** The most stable arrangement of the terminated Ti_2_NT_x_ layers by oxygen atoms for two adjacent Ti_2_N layers. The solid line denotes the lateral size of the supercell (top view). **b** Interlayer binding energy curve of Ti_2_NO_2_ layers. The interlayer binding energy decreases as the number of layers increases, reaching a value of − 16.6 meV/atom for an infinite number of layers. The black line fitted to the data is presented, and the extrapolated value for an infinite number of layers is indicated

D_layer_ and d_interlayer_ indicate the vertical distance between the Ti atoms within the same layer and the Ti atoms from adjacent layers (see Fig. 4a), respectively

The interlayer binding energy is defined as:

2 $$E_{b}^{n} = \frac{{E_{tot}^{n} \left( {Ti_{2} NO_{2} } \right) - nE_{tot}^{1L} \left( {Ti_{2} NO_{2} } \right)}}{N},$$

where $E_{tot}^{n}$ and $E_{tot}^{1L}$ are the total energies of the n-layered and one monolayer of Ti_2_NO_2_, respectively. One can easily see that the interlayer binding energy is simply the difference in cohesive energies (*E*_*coh*_) of n-layered and monolayer structures:

3 $$E_{b}^{n} = \frac{{E_{coh}^{n} \left( {Ti_{2} NO_{2} } \right) - nE_{coh}^{1L} \left( {Ti_{2} NO_{2} } \right)}}{N},$$

where $E_{coh}^{n}$ is defined as:

4 $$E_{coh}^{n} \left( {Ti_{2} NO_{2} } \right) = E_{tot}^{n} \left( {Ti_{2} NO_{2} } \right) - 2nE_{atm} \left( {Ti} \right) - nE_{atm} \left( N \right) - 2nE_{atm} \left( O \right),$$

where $E_{tot}^{n}$ is the total energy of the n-layered Ti_2_NO_2_, $E_{atm}^{{}}$ is the free energy of the given atom (isolated atom), and *n* and *N* stand for the number of layers and the total number of atoms in the given system, respectively.

Moreover, in case of the Ti_2_NT_x_ the experiments have proved that the majority of the groups bonded to Ti in Ti_2_N were O- groups rather than F- or OH- (see, Fig. 2l). Prompted by these results, we considered the fully functionalized Ti_2_NT_x_ MXene layers to be functionalized by oxygen atoms (Ti_2_NO_2_), as presented in Fig. 4a. We examined the stability of Ti_2_NO_2_ MXenes that contained a few layers (up to five layers) as a function of the number of the layers, by calculating the interlayer binding energy, which reflects the strength of the interaction forces between the adjacent layers. The greater the negative value of the interlayer binding energy the more the system is stable. Our studies from first principles revealed that the stability of the TiN_2_O_2_ increased with an increasing number of layers in the sample (see Fig. 4b). Specifically, the difference in interlayer binding energy between five layers and one monolayer was equal to − 13.2 meV atom^−1^, indicating that the 5L structure was more stable than the monolayer system.

Our results showed that the stabilization of the TiN_2_O_2_ can be enhanced by adding layers to the structure, and the interlayer binding energy can reach a limit of − 16.6 meV per atom for an infinite number of layers. In other words, the weak van der Waals forces stabilize TiN_2_O_2_ material containing a few layers. This result indicates that it is experimentally easier to obtain thicker samples than monolayer ones.

### Results of in vitro studies, mode of action analysis, and stability features

**Fig. 5:**
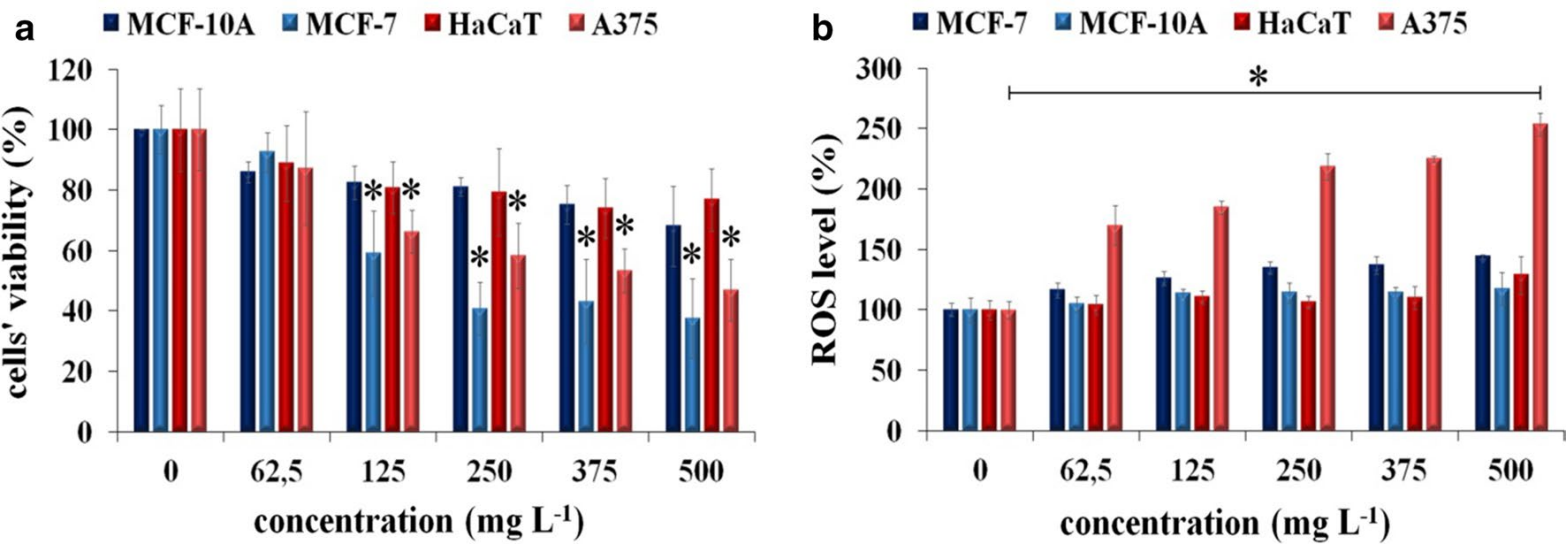
**a** The results of MTT assay after 24 h exposure of A549, MRC-5, A375, and HaCaT cells to increasing concentrations of multilayered 2D sheets of Ti_2_NT_x_ MXene. **b** Production of reactive oxygen species (ROS) during incubation with various concentrations of 2D sheets of Ti_2_NT_x_ MXene. Student’s *t* test, *α *= 0.005, *n *= 3; *n* number of independent experiments

The biocompatibility of multilayered 2D sheets of Ti_2_NT_x_ MXene was tested on HaCaT and A375 human skin cells as well as on MCF-10A and MCF-7 human breast cells. The cellular cultures were exposed to nanomaterial dispersions in complete DMEM or DMEM F-12 (dependent on the tested cell line) medium (at 0–500 µg mL^−1^). The incubation of normal cells with 2D Ti_2_NT_x_ resulted in a slight loss of cell viability in the whole range of applied concentrations (Fig. 5a). For comparison, cancer cells treated with the same nanomaterial showed a significant loss of their viability at 250 µg mL^−1^ (cellular survival below 70%). It is worth noting that dose-related cytotoxicity was also indicated for both nonmalignant cell lines, but not in such a manner as for the cancerous cells. The probable causes of the observed effect of the enhanced cytotoxicity of MXene towards cancerous cells as compared to non-malignant ones were an altered mechanism of subcellular internalization and oxidative stress phenomena resulting in increased ROS generation. Our previous studies on the cytotoxicity of MXenes in the form of two-dimensional titanium carbides [19] indicated that ROS generation was the main mechanism of those nanomaterials’ action. Some other studies have also indicated that oxidative stress is the most probable cause of two-dimensional materials’ cyto- and ecotoxicity [48–50]. The intracellular ROS production in all the tested cell lines is presented in Fig. 5b. Therefore, the results obtained confirmed that intercellular ROS release occurred mostly in A375 cells, where there was a statistically significant difference between the controls and groups treated with 2D Ti_2_NT_x_. For each normal cell line, as well as for MCF-7, the DCF fluorescence was maintained at the same level compared to controls. The results obtained for MCF-7 suggest different mechanisms of the nanomaterial’s toxicity, which can be potentially associated with the cellular metabolism and chemical composition of the cellular membrane.

**Fig. 6:**
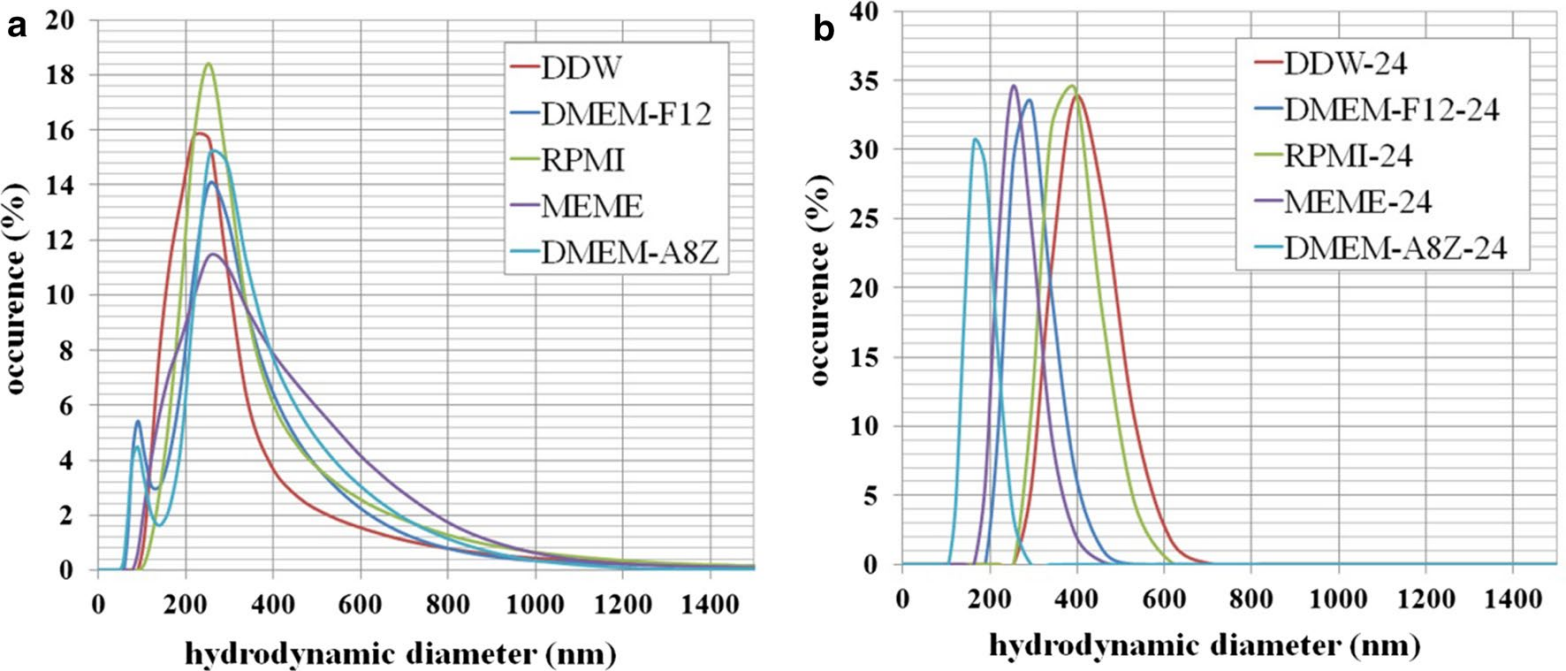
Distributions of the hydrodynamic diameters obtained for multilayered 2D sheets of Ti_2_NT_x_ MXene suspended in double-distilled water (DDW) as well as cultivation media applied for the in vitro studies, for incubation time t = 0 and t = 24 h

It was also important in our studies to check the stability of the nanomaterials in the in vitro studies because rapid agglomeration is a typical phenomenon observed in materials’ dispersions in cells cultivation media. Figure 6 shows the distributions of the hydrodynamic diameters obtained for multilayered 2D sheets of Ti_2_NT_x_ MXene suspended in double distilled water (DDW) as well as the cultivation media typically used for the in vitro studies. Small differences were noticed in the case of DMEM media in relation to RPMI and MEME in case of incubation time = 0 (Fig. 6a). For these media, a small peak was seen around 100 nm, which does not influence the major peak located ca. 550 nm (see also data in Tab. 3). The same peak was present for 2D sheets of Ti_2_NT_x_ MXene suspended in DDW. What is interesting, after incubation of madia for 24 h, the maxima of the peaks shifted for c.a., 60% toward lower sizes and became mode sharp in relation to peaks from time = 0 (see Fig. 6b and Table 3). This suggest that the cultivation media can help in gaining better stability of the 2D flakes. The stability analysis also included zeta potential studies. The obtained results are presented in Table 3. The primary zeta potential value of the 2D sheets of Ti_2_NT_x_ MXene suspended in DDI water was − 21.4 mV. It is worth noting that when the material was placed in cultivation media, the nutrients adsorbed on its surface, changing its original zeta potential. Indeed, the zeta potential of the 2D Ti_2_NT_x_ suspended in all cultivation media changed to a range from − 7.8 to − 8.6 mV and stabilized within this range of values. When taking into consideration the stability of the hydrodynamic diameter of the tested 2D flakes, it can be thus concluded that the analyzed 2D flakes are stable in the in vitro analysis environment, and it does not influence the results of the investigation.

**Table 3:** Stability analysis results obtained for multilayered 2D sheets of Ti_2_NT_x_ MXene suspended in double distilled water (DDW) water as well as cultivation media applied for the in vitro studies, for incubation time t = 0 and t = 24 h

Cultivation medium	DDW	DMEM-F12	RPMI	MEME	DMEM-A8Z
Zeta potential after t = 0 (mV)	− 21.3 ± 0.1	− 7.6 ± 0,3	− 8.4 ± 0.6	− 8.3 ± 0.3	− 8.0 ± 0.6
Zeta potential after t = 24 (mV)	− 21.4 ± 0.2	− 8.0 ± 0.2	− 8.6 ± 1.0	− 7.9 ± 0,1	− 9.1 ± 0.8
Hydrodynamic diameter after t = 0 (nm)	564 ± 24	611 ± 21	542 ± 12	595 ± 16	518 ± 13
Hydrodynamic diameter after t = 24 (nm)	383 ± 44	293 ± 11	277 ± 54	325 ± 86	177 ± 25

**Fig. 7:**
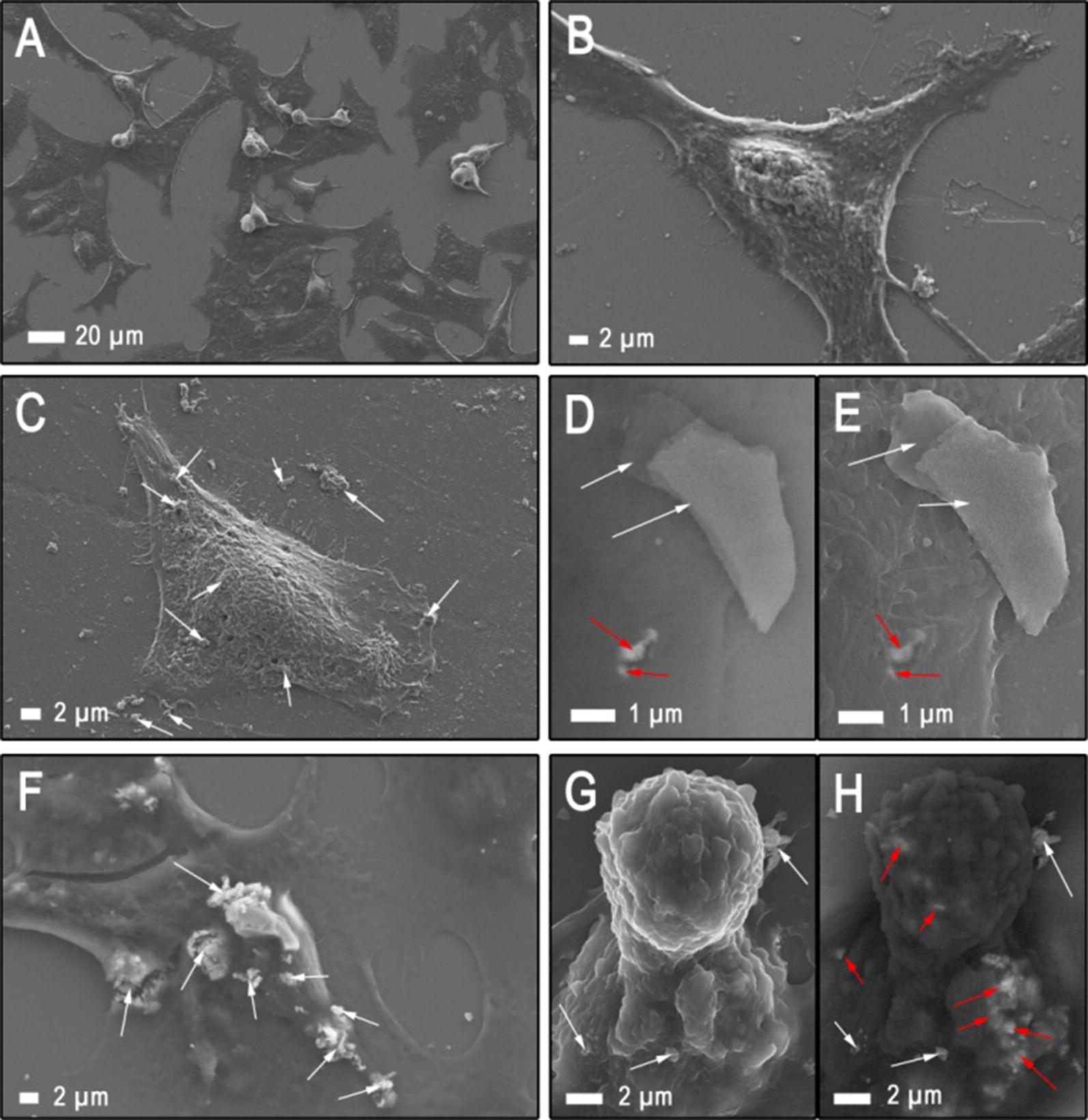
Representative SEM images obtained for (**a**, **b**) reference A375 cell line, and A375 cells treated with multilayered 2D sheets of Ti_2_NT_x_ MXene at a concentration of (**c–e**) 62.5 mg L^−1^ and (**f–h**) 500 mg L^−1^. SEM analysis was performed with SE mode (**a–g**) as well as AsB (**d**, **h**). 2D Ti_2_N sheets internalized by A375 cells were marked with red arrows, whereas those only attached to the cells’ surface were marked with white arrows

**Fig. 8:**
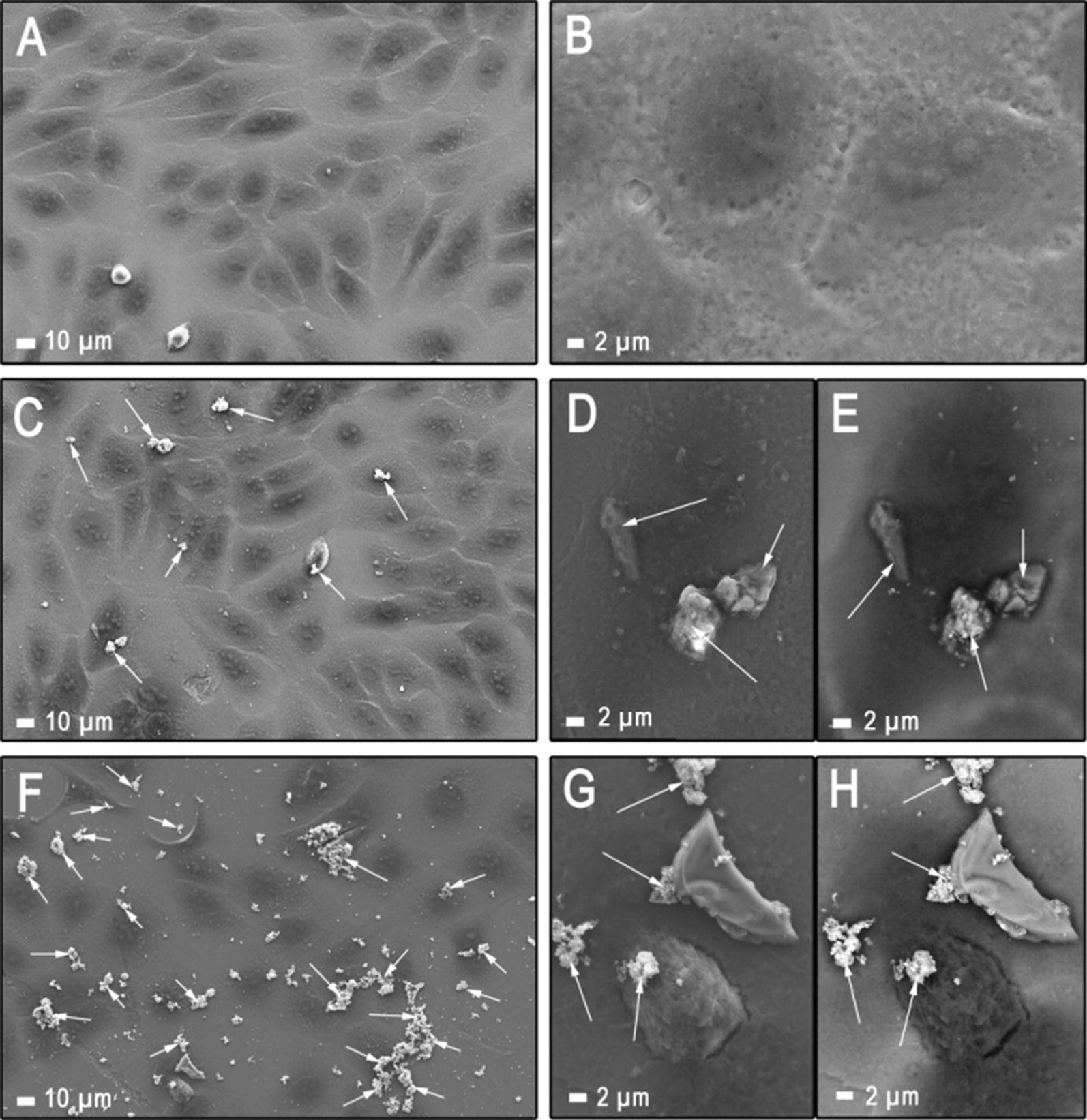
Representative SEM images obtained for (**a**, **b**) reference HaCaT cell line, and HaCaT cells treated with multilayered 2D sheets of Ti_2_NT_x_ MXene at a concentration of (**c**, **d**, **e**) 62.5 mg L^−1^ and (**f**, **g**, **h**) 500 mg L^−1^. SEM analysis was performed with SE mode (**a–d**, **g**) as well as ASB (**e**, **h**). 2D Ti_2_NT_x_ sheets attached to the surface of the HaCaT cells were marked with white arrows

In order to observe the material’s internalization and its potential biodistribution inside the cells, scanning electron microscopy (SEM) combined with an energy-selective backscattered (ESB) detector for the imaging of compositional differences was applied. Figure 7 shows examples of microscopic images of A375 after exposure to multilayered 2D Ti_2_NT_x_, while the nanomaterial’s uptake by HaCaT is presented in Fig. 8. The applied concentrations of the MXene were set at 62.5 μg mL^−1^ and 500 μg mL^−1^, respectively. The SEM data clearly indicates that skin cancerous cells internalize 2D Ti_2_NT_x_ flakes more efficiently (Fig. 7) comparing to nonmalignant cells (Fig. 8). It also should be stressed that exposure to Ti_2_NT_x_ results in much lower morphological changes in the case of HaCaT cells compared to the A375 line at each concentration applied for SEM investigations.

## Conclusion

2D Ti_2_NT_x_ MXene is expected to pave the way for MXenes to be used in many effective applications. While 2D sheets of Ti_3_C_2_T_x_ or Ti_2_CT_x_ MXenes have already been widely studied with regard to their cytotoxicity, research on 2D Ti_2_NT_x_ has not been focused on biologically-related studies. Here, we have undertaken the challenge of investigating the cytotoxic properties of the Ti_2_NT_x_ MXene phase after classical acidic aluminum etching of the Ti_2_AlN MAX phase and further two-step delamination into multilayered 2D nano-flakes. It should be noted that the first experimental studies carried out on 2D Ti_2_NT_x_ monolayers revealed large difficulties in their synthesis and confirmed their low stability realized through rapid oxidation and decomposition. Our theoretical calculations showed a higher susceptibility towards the formation of multilayered Ti_2_NT_x_ structures during the preparation process in comparison to single-layered structures. Therefore, the Ti_2_AlN MAX phase was successfully etched into Ti_2_NT_x_ MXene and further delaminated to obtain multilayered 2D nano-sheets of the appropriate stability for biological studies. The in vitro tests were carried out using two pairs of normal and cancerous cell lines. Additionally, the potential mode of action of 2D Ti_2_NT_x_ was investigated using a reactive oxygen test as well as SEM observations. Specifically, this study reveals that:

- Theoretical calculations show the possibility of bypassing the problem of instability of single-layered 2D Ti_2_NT_x_ MXene i.e., higher likelihood of forming multilayered Ti_2_NT_x_ structures during the preparation process in comparison to single-layered structures,
- 2D multilayered sheets of Ti_2_NT_x_ MXene can be thus successfully obtained using classic etching and delamination,
- The multilayered 2D sheets of Ti_2_NT_x_ show higher toxicity towards cancerous cell lines (MCF-7and A365) in comparison to normal ones,
- The decrease in the cells’ viability is dose-dependent,
- 2D Ti_2_NT_x_ is not toxic towards non-malignant cells (MCF-10A and HaCaT),
- The identified mechanisms of toxicity are the generation of reactive oxygen species as well as the 2D sheets’ internalization.

The results of the present study provide the principal knowledge to date regarding the toxicity and potential anticancer properties of the delaminated 2D multilayered sheets of Ti_2_N MXene. We also reveal that the 2D Ti_2_N in its multilayered form exhibits fair stability, so can be applied in in vitro studies. These results show promise for its future application in biotechnology and nanomedicine.

## Data Availability

Authors declare availability of data and materials that were used for this study at WUT.

## References

[1] Novoselov KS, Geim AK, Morozov SV, Jiang D, Zhang Y, Dubonos SV, Grigorieva IV, Firsov AA (2004). Electric field effect in atomically thin carbon films. Science.

[2] Manzeli S, Ovchinnikov D, Pasquier D, Yazyev OV, Kis A (2017). 2D transition metal dichalcogenides. Nat Rev Mater..

[3] Pacilé D, Meyer JC, Girit CO, Zettl A (2008). The two-dimensional phase of boron nitride: few-atomic-layer sheets and suspended membranes. Appl Phys Lett..

[4] Franzen HF, Smeggil J, Conard BR (1967). The group IV di-transition metal sulfides and selenides. Mater Res Bull..

[5] Yin J, Wu B, Wang Y, Li Z, Yao Y, Jiang Y, Ding Y, Xu F, Zhang P (2018). Novel elastic, lattice dynamics and thermodynamic properties of metallic single-layer transition metal phosphides: 2H-M _2_P (Mo_2_P, W_2_P, Nb_2_P and Ta_2_P). J Phys.

[6] Ma R, Sasaki T (2010). Nanosheets of oxides and hydroxides: ultimate 2D charge-bearing functional crystallites. Adv Mater..

[7] Treacy MMJ, Rice SB, Jacobson AJ, Lewandowski JT (1990). Electron microscopy study of delamination in dispersions of the perovskite-related layered phases K[Ca2Nan-3NbnO3n-1]: evidence for single-layer formation. Chem Mater..

[8] Colson JW, Dichtel WR (2013). Rationally synthesized two-dimensional polymers. Nat Chem..

[9] Molle A, Goldmerger J, Houssa M, Xu Y, Zhang S-C, Akinwande D (2017). Buckled two-dimensional Xene sheets. Nat Mater..

[10] Naguib M, Kurtoglu M, Presser V, Lu J, Niu J, Heon M, Hultman L, Gogotsi Y, Barsoum MW (2011). Adv Mater..

[11] Anasori B, Lukatskaya MR, Gogotsi Y (2017). 2D metal carbides and nitrides (MXenes) for energy storage. Nat Rev Mater..

[12] Wozniak J, Petrus M, Cygan T, Jastrzębska A, Wojciechowski T, Ziemkowska W, Olszyna A (2019). Silicon carbide matrix composites reinforced with two-dimensional titanium carbide—manufacturing and properties. Ceram Intern..

[13] Guo J, Legum B, Anasori B, Wang K, Lelyukh P, Gogotsi Y, Randall CA (2018). Cold sintered ceramic nanocomposites of 2D MXene and zinc oxide. Adv Mater..

[14] Feia M, Lin R, Lu Y, Zhang X, Bian R, Cheng J, Luo P, Xu C, Cai D (2017). MXene-reinforced alumina ceramic composites. Ceram Intern..

[15] Rozmysłowska-Wojciechowska A, Wojciechowski T, Ziemkowska W, Chlubny L, Olszyna A, Jastrzębska AM (2019). Surface interactions between 2D Ti3C2/Ti2C MXenes and lysozyme. Appl Surf Sci..

[16] Jastrzębska AM, Karwowska E, Wojciechowski T, Ziemkowska W, Rozmysłowska A, Chlubny L, Olszyna A (2018). The atomic structure of Ti_2_C and Ti_3_C_2_ MXenes is responsible for their antibacterial activity toward *E. coli* bacteria. J Mater Eng Perform..

[17] Jastrzębska A, Karwowska E, Basiak D, Zawada A, Ziemkowska W, Wojciechowski T, Jakubowska D, Olszyna A (2017). Biological activity and bio-sorption properties of the Ti_2_C studied by means of zeta potential and SEM, accepted for publication in Int. J Electrochem Sci..

[18] Rasool K, Helal M, Ali A, Ren CE, Gogotsi Y, Mahmoud KA (2016). Antibacterial activity of Ti3C2Tx MXene. ACS Nano.

[19] Szuplewska A, Kalinowska D, Dybko A, Jastrzębska AM, Wojciechowski T, Rozmysłowska A, Chudy M, Grabowska-Jadach I, Ziemkowska W, Brzózka Z, Olszyna A (2019). 2D Ti_2_C (MXene) as a novel, highly efficient agent for photothermal therapy. Mater Sci Eng C.

[20] Zhang Y, Wang L, Zhang N, Zhou Z (2018). Adsorptive environmental applications of MXene nanomaterials: a review. RSC Adv..

[21] Deysher G, Sin S, Gogotsi Y, Anasori B (2018). Oxidized 2D titanium carbide MXene. Mater Today.

[22] Hope MA, Forse AC, Griffith KJ, Lukatskaya MR, Ghidiu M, Gogotsi Y, Grey CP (2016). NMR reveals the surface functionalisation of Ti3C2 MXene. Phys Chem Chem Phys.

[23] Hu T, Li Z, Hu M, Wang J, Hu Q, Li Q, Wang X (2017). Chemical Origin of Termination-Functionalized MXenes: Ti3C2T2 as a Case Study. J Phys Chem C.

[24] Lotfi R, Naguib M, Yilmaz DE, Nanda J, van Duin ACT (2018). A comparative study on the oxidation of two-dimensional Ti3C2 MXene structures in different environments. J Mater Chem A.

[25] Li X, Wang C, Cao Y, Wang G (2018). Functional MXene materials: progress of their applications. Chem Asian J.

[26] Anasori B, Lukatskaya MR, Gogotsi Y (2017). 2D metal carbides and nitrides (MXenes) for energy storage. Nat Rev Mater..

[27] Kumar H, Frey NC, Dong L, Anasori B, Gogotsi Y, Shenoy VB (2017). Tunable magnetism and transport properties in nitride MXenes. ACS Nano.

[28] Urbankowski P, Anasori B, Makaryan T, Er D, Kota S, Walsh PL, Zhao M, Shenoy VB, Barsoum MW, Gogotsi Y (2016). Synthesis of two-dimensional titanium nitride Ti_4_N_3_ (MXene). Nanoscale.

[29] Tsai H-S, Hsu C-H, Chi C-C, Wang Y-C, Liu F-W, Tang S-Y, Tsai C-J, Ouyang H, Chueh Y-L, Liang J-H (2018). Non-layered Ti2 N synthesized by plasma process for the anodes of lithium-ion batteries. Inorg Chem Front..

[30] Wriedt HA, Murray JL (1987). Bull. Alloy Phase Diagrams.

[31] Gao G, Ding G, Li J, Yao K, Wu M, Qianb M (2016). Monolayer MXenes: promising half-metals and spin gapless semiconductors. Nanoscale.

[32] Soundiraraju B, George BK (2017). Two-dimensional titanium nitride (Ti2N) MXene: synthesis, characterization, and potential application as surface-enhanced raman scattering substrate. ACS Nano.

[33] Yang C, Tang Y, Tian Y, Luo Y, Din MF, Yin X, Que W (2018). Flexible nitrogen-doped 2D titanium carbides (MXene) films constructed by an ex situ solvothermal method with extraordinary volumetric capacitance. Adv Energy Mat..

[34] Hohenberg P, Kohn W (1964). Inhomogeneous electron gas. Phys Rev..

[35] Kohn W, Sham LJ (1965). Self-consistent equations including exchange and correlation effects. Phys Rev..

[36] Perdew JP, Burke K, Ernzerhof M (1996). Generalized gradient approximation made simple. Phys Rev Lett.

[37] Kresse G, Hafner J (1993). Ab initio. Phys Rev B.

[38] Kresse G, Muller J (1996). Effciency of ab initio total energy calculations for metals and semiconductors using a plane-wave basis set. Comput Mater Sci..

[39] Blochl PE (1994). Projector augmented-wave method. Phys Rev B.

[40] Kresse G, Joubert D (1999). From ultrasoft pseudopotentials to the projector augmented-wave method. Phys Rev B.

[41] Grimme S (2006). Semiempirical gga-type density functional constructed with a long-range dispersion correction. J Comput Chem.

[42] Monkhorst HJ, Pack JD (1976). Special points for brillouin-zone integrations. Phys Rev B.

[43] Dudarev SL, Botton GA, Savrasov SY, Humphreys CJ, Sutton AP (1998). Electron-energy-loss spectra and the structural stability of nickel oxide: an lsda + u study. Phys Rev B.

[44] Zhang N, Hong Y, Yazdanparast S, Zaeem MA (2018). Superior structural, elastic and electronic properties of 2d titanium nitride MXenes over carbide MXenes: a comprehensive first principles study. 2D Mater..

[45] Milowska K, Birowska M, Majewski JA (2011). Mechanical, electrical, and magnetic properties of functionalized carbon nanotubes. AIP Conf Proc.

[46] Milowska K, Birowska M, Majewski JA (2009). Ab initio study of functionalized carbon nanotubes. Acta Phys Pol A.

[47] Xie Y, Kent PRC (2013). Hybrid density functional study of structural and electronic properties of functionalized tin + 1Xn (x = C, n) monolayers. Phys Rev B.

[48] Feng L, Yang D, Gai S, He F, Yang G, Yang P, Lin J (2018). Single bismuth tungstate nanosheets for simultaneous chemo-, photothermal, and photodynamic therapies mediated by near-infrared light. Chem Eng J.

[49] Gurunathan S, Kang MH, Jeyaraj M (2019). Differential cytotoxicity of different sizes of graphene oxide nanoparticles in leydig (TM3) and sertoli (TM4) cells. Nanomaterials.

[50] Yuan P, Zhou Q, Hu X (2019). The phases of WS_2_, nanosheets influence uptake, oxidative stress, lipid peroxidation, membrane damage, and metabolism in algae. Environ Sci Technol.

